# Survey and analysis of the nutritional status in hospitalized patients with malignant gastric tumors and its influence on the quality of life

**DOI:** 10.1007/s00520-019-04803-3

**Published:** 2019-05-03

**Authors:** Zeng Qing Guo, Jia Mi Yu, Wei Li, Zhen Ming Fu, Yuan Lin, Ying Ying Shi, Wen Hu, Yi Ba, Su Yi Li, Zeng Ning Li, Kun Hua Wang, Jing Wu, Ying He, Jia Jun Yang, Cong Hua Xie, Xin Xia Song, Gong Yan Chen, Wen Jun Ma, Su Xia Luo, Zi Hua Chen, Ming Hua Cong, Hu Ma, Chun Ling Zhou, Wei Wang, Qi Luo, Yong Mei Shi, Yu Mei Qi, Hai Ping Jiang, Wen Xian Guan, Jun Qiang Chen, Jia Xin Chen, Yu Fang, Lan Zhou, Yong Dong Feng, Rong Shao Tan, Tao Li, Jun Wen Ou, Qing Chuan Zhao, Jian Xiong Wu, Li Deng, Xin Lin, Liu Qing Yang, Mei Yang, Chang Wang, Chun Hua Song, Hong Xia Xu, Han Ping Shi, The Investigation on the Nutrition Status and Clinical Outcome of Common Cancers (INSCOC) Group

**Affiliations:** 1 Department of Medical Oncology, Fujian Cancer Hospital, Fujian Medical University Cancer Hospital, Fuzhou, 350014 Fujian China; 2 Cancer Center of the First Hospital of Jilin University, Changchun, 130021 Jilin China; 3 Cancer Center, Renmin Hospital of Wuhan University, Wuhan, 430060 Hubei China; 4 Department of Gastrointestinal Surgery, Affiliated Tumor Hospital of Guangxi Medical University, Nanning, 530021 Guangxi China; 5 Department of Surgery, The First Affiliated Hospital of Sun Yat-Sen University, Guangzhou, 510080 Guangdong China; 6 Department of Clinical Nutrition, West China Hospital of Sichuan University, Chengdu, 610041 Sichuan China; 7 Department of Gastrointestinal Oncology, National Clinical Research Center for Cancer, Tianjin Key Laboratory of Cancer Prevention and Therapy, Tianjin Medical University Cancer Institute and Hospital, Tianjin, 300060 China; 8 Department of Nutrition and Metabolism of Oncology, Affiliated Provincial Hospital of Anhui Medical University, Hefei, 230031 Anhui China; 9 Department of Clinical Nutrition, The First Hospital of Hebei Medical University, Shijiazhuang, 050031 Hebei China; 10 Department of Gastrointestinal Surgery, Institute of Gastroenterology, The First Affiliated Hospital of Kunming Medical University, Kunming, 650032 Yunnan China; 11 Department of Clinical Nutrition, The First People’s Hospital of Kashi, Xinjiang, 844000 China; 12 Department of Clinical Nutrition, Chongqing General Hospital, Chongqing, 400014 China; 13 Department of Colorectal and Anal Surgery, Huizhou Municipal Central Hospital, Huizhou, 516001 Guangdong China; 14 Department of Radiation and Medical Oncology, Zhongnan Hospital of Wuhan University, Wuhan, 430071 Hubei China; 15 Department of Oncology, Xingtai People’s Hospital, Hebei Medical University, Xingtai, 054031 Hebei China; 16 The First Department of the Tumor Hospital of Harbin Medical University, Harbin, 150085 Heilongjiang China; 17 Department of Nutrition, Guangdong General Hospital, Guangdong Academy of Medical Sciences, Guangzhou, 510080 Guangdong China; 18 Department of Oncology, Affiliated Cancer Hospital of Zhengzhou University and Henan Cancer Hospital, Zhengzhou, 450008 Henan China; 19 Department of General Surgery, Xiangya Hospital, Central South University, Changsha, 410008 Hunan China; 20 Comprehensive Oncology Department, Cancer Hospital, Chinese Academy of Medical Sciences, Beijing, 100021 China; 21 Department of Oncology, Affiliated Hospital of Zunyi Medical University, Zunyi, 563000 Guizhou China; 22 The Fourth Affiliated Hospital, Harbin Medical University, Harbin, 150001 Heilongjiang China; 23 Cancer Center, The First People’s Hospital of Foshan, Foshan, 528000 Guangdong China; 24 Department of Gastrointestinal Tumor Surgery, The First Affiliated Hospital of Xiamen University, Xiamen, 361003 Fujian China; 25 Department of Nutrition, Ruijin Hospital, Shanghai Jiao Tong University School of Medicine, Shanghai, 200025 China; 26 Department of Nutrition, Tianjin Third Central Hospital, Tianjin, 300170 China; 27 Department of Surgery, The First Affiliated Hospital of Jinan University, Guangzhou, 510632 Guangdong China; 28 Department of General Surgery, Nanjing Drum Tower Hospital, The Affiliated Hospital of Nanjing University Medical School, Nanjing, 210008 Jiangsu China; 29 Department of Gastrointestinal Surgery, First Affiliated Hospital of Guangxi Medical University, Nanning, 530021 Guangxi China; 30 Department of Radiation and Medical Oncology, People’s Hospital of Guangxi Zhuang Autonomous Region, Nanning, 530021 Guangxi China; 31 Department of Clinical Nutrition, Peking University Cancer Hospital and Institute, Beijing, 100142 China; 32 Department of Nutrition, Tumor Hospital of Yunnan Province, Third Affiliated Hospital of Kunming Medical College, Kunming, 650118 Yunnan China; 33 Department of Surgery, Tongji Hospital, Tongji Medical College, Huazhong University of Science and Technology, Wuhan, 430030 China; 34 Department of Nutrition, Guangzhou Red Cross Hospital, Guangzhou, 510220 Guangdong China; 35 Department of Radiotherapy, , School of Medicine, Sichuan Cancer Hospital & Institute, Sichuan Cancer Center, University of Electronic Science and Technology of China, Chengdu, 610041 Sichuan China; 36 Department of Clinical Nutrition, Clifford Hospital, Guangzhou University of Chinese Medicine, Guangzhou, 510632 Guangdong China; 37 Department of Digestive Diseases, Xijing Hospital, Fourth Military Medical University, Xi’an, 710032 Shanxi China; 38 Department of Hepatobiliary Surgery, National Cancer Center/Cancer Hospital, Chinese Academy of Medical Sciences and Peking Union Medical College, Beijing, 100021 China; 39 Department of Nutrition, Daping Hospital & Research Institute of Surgery, Third Military Medical University, Chongqing, 400042 China; 40 Department of Gastrointestinal Surgery/Clinical Nutrition, Beijing Shijitan Hospital, Capital Medical University, No.10 Tieyi Road, Haidian District, Beijing, 100038 China; 41 Department of Epidemiology, College of Public Health, Zhengzhou University, Zhengzhou, 450001 Henan China

**Keywords:** Gastric cancer, Patient-generated subjective global assessment (PG-SGA), Malnutrition, Quality of life

## Abstract

**Background/objectives:**

The assessment of nutritional status and the quality of life in patients with gastric cancer has become one of the important goals of current clinical treatment. The purpose of this study was to assess the nutritional status in hospitalized gastric cancer patients by using patient-generated subjective global assessment (PG-SGA) and to analyze the influence of nutritional status on the patients’ quality of life (QOL).

**Methods:**

We reviewed the pathological diagnosis of gastric cancer for 2322 hospitalized patients using PG-SGA to assess their nutritional status and collected data on clinical symptoms, the anthropometric parameters (height, weight, body mass index (BMI), mid-arm circumference (MAC), triceps skin-fold thickness (TSF), and hand-grip strength (HGS). We also collected laboratory data (prealbumin, albumin, hemoglobin) within 48 h after the patient was admitted to the hospital. The 30-item European Organization for Research and Treatment of Cancer Core Quality of Life Questionnaire (EORTC QLQ-C30) was used for QOL assessment in all patients.

**Results:**

By using PG-SGA, we found 80.4% of the patients were malnourished (score ≥ 4) and 45.1% of the patients required urgent nutritional support (score ≥ 9). In univariate analysis, old age (> 65 years, *p* < 0.001), female (*p* = 0.007), residence in a village (*p* = 0.004), a lower level of education (*p* < 0.001), and self-paying (*p* < 0.001) were indicated as risk factors of patients with gastric cancer to be suffering from severe malnutrition. There was a negative correlation between PG-SGA and various nutritional parameters (*p* < 0.05). The quality of life was significantly different in gastric cancer patients with different nutritional status (*p* < 0.01).

**Conclusion:**

Malnutrition of hospitalized patients with gastric cancer in China is common and seriously affects the patients’ quality of life. The nutritional status should be evaluated in a timely manner and reasonable nutritional intervention should be provided as soon as possible. The PG-SGA was fit for using as a clinical nutrition assessment method, being worthy of clinical application.

## Introduction

At present, the incidence of cancer and the rate of mortality are still rising and are a major disease threat to human life and health. The incidence of and mortality from gastric cancer is second in morbidity and mortality behind only lung cancer in China [1]. Studies have reported that 50~90% of patients with malignant tumors have weight loss and suffer from malnutrition [2]. This is especially true in patients with head and neck cancer and malignant digestive tract tumors. The high incidence of malnutrition in gastric cancer patients is due to the tumor location [3, 4]. About 20% of patients die due to malnutrition and related complications, not from the malignant tumor itself [5–7]. The quality of life between the patients in good nutrition and in malnutrition is different, so the nutrition assessment of the patients should be paid more attention to, in order to improve the nutritional status and the quality of life of the patients. However, no nutritional assessment method is currently available that can be considered the gold standard nor is there a consensus on which assessment would be the best option, and there are few studies of nutritional assessment of patients with gastric cancer. The purpose of this study was to evaluate the nutritional status of hospitalized patients with gastric cancer and to analyze the influence of their nutritional status on their quality of life. The long-term goal is to provide an effective and appropriate nutrition assessment tool for guiding the clinical treatment of these patients.

## Materials/subjects and methods

### Materials

A multi-center, cross-sectional observational study was carried out. It was one part of the Investigation on Nutritional Status and its Clinical Outcomes of Common Cancers (INSCOC). The INSCOC is a nationwide cross-sectional survey on the correlation between nutritional status and clinical outcome in patients with malignant tumors. It was initiated and implemented by the Chinese Cancer Society Cancer nutrition and support Specialized Committee. A total of 2322 gastric cancer patients were included from January 2012 to August 2016 at several tertiary public hospitals in China. Inclusion criteria were as follows: (1) an age of 18 to 90 years, conscious, no communication disorders, and can cooperate with relevant inspection; (2) a histologic diagnosis of gastric cancer; (3) only patients in the hospital many times for the same case can take part in this survey; (4) there are complete medical history records and follow-up data; (5) the patient and family voluntarily participate in this study. Exclusion criteria were as follows: (1) AIDS patients or organ transplant patients; (2) patient in a critical condition and difficult to assess; (3) patients refuse or do not cooperate with a questionnaire. This study was approved by the Ethics Committee of each participating hospital and complied with the Declaration of Helsinki.

### Assessment method

PG-SGA was developed by Ottery [8]. It includes patients’ self-reported sections (body weight, eating conditions, symptoms, activities, and physical function) and a medical personnel assessment part (nutrition-related disease state, metabolic state, physical examination) in seven domains. The sum of scores obtained in each domain is divided into quantitative and qualitative evaluations. Quantitative evaluation results are scores of 0–3 (well-nourished/suspicious malnutrition), 4–8 (moderate malnutrition), and ≥ 9 (severe malnutrition). Patients scoring 4 to 8 points require nutritional intervention by a dietitian with a clinical symptom survey. Patients scoring ≥ 9 points are in great need of symptom management and nutrition intervention before anti-tumor treatment.

NRS2002 is a nutritional risk screening tool recommended by the European Society for Parenteral and Enteral Nutrition (ESPEN) [9], based on 128 randomized controlled trials. It includes three parts [10]: a disease score (0–3), nutrition score (0–3), and age (70 years or older has a score of 1), the sum score of nutritional risks (score of 0 to 7). A score of ≥ 3 means there is a nutritional risk and the patient should start on a nutritional treatment plan. Scores of less than 3 can be regarded as no nutritional risk, but patients still need to be screened weekly during hospitalization.

The 30-item European Organization for Research and Treatment of Cancer Core Quality of Life Questionnaire (EORTCQLQ-C30) is a systematic evaluation approach for determining the quality of life of cancer patients. The Chinese version of EORTC QLQ-C30 V3.0 has been proven to be valid, reliable, and clinically relevant [11]. It includes 30 subjects divided into five categories defining functions (physical function, role function, emotional function, cognitive function, and social function), three categories qualifying symptoms (fatigue, nausea and vomiting, pain), six single measurement subjects (difficulty in breathing, insomnia, loss of appetite, constipation, diarrhea, economic difficulties), and one score for the overall quality of life. Scores for the functional or symptom categories and for the single measurement subjects are calculated by a linear transformation of raw scores into a 0 to 100 score. Scores of 100 represent the best outcomes on the QLQ-C30 functional categories and the worst outcomes on the QLQ-C30 symptom categories. Weight (W) was measured to the nearest 0.1 kg by an electronic scale and height (H) was measured using a portable vertical stadiometer [12]. Patients stood upright on the center of the scale with arms extended laterally, barefoot, and wearing light clothing. From the measurements of W and H, the body mass index (BMI) was calculated: BMI (kg/m^2^) = weight (kg)/height (m)^2^. Mid-arm circumference (MAC) and triceps skin-fold thickness (TSF) were measured on the non-dominant arm according to Frisancho [13]. The hand-grip strength (HGS) method measurement can be referenced to Schlüssel [14]. All the measurements were performed in triplicate, where the final result was the average of the values.

Fasting blood samples for assessment of albumin, prealbumin, and hemoglobin were obtained within 48 h after the patients were admitted to the hospital. Laboratory data were measured by standard laboratory methods.

### Methods

All the measurements were performed by trained researchers. An adopted unified design and unified questionnaires were administered within 48 h after admission by physicians and/or specialist nutrition nurses who had received standardized training. The nutritional status was evaluated by PG-SGA, and the quality of life assessed by the EORTC QLQ-C30. Related data were collected, recorded, and checked. The database was then finally determined.

### Statistical analysis

Statistical analysis was carried out using SPSS version 21 (SPSS Institute, Inc.). Descriptive statistics (means, standard deviations, and frequencies) were expressed. The degree of relationship among these factors and the PG-SGA scores was statistically evaluated using the *t* test, ANOVA test, and correlation analyses. Statistical significance was reported at the *p* < 0.05 level.

## Results

A total of 2322 hospitalized patients with gastric cancer were analyzed through this study. There were 1628 males and 694 females, with a mean age of 62 years, ranging from 25 to 90 years old. According to the PG-SGA, 19.6% of patients were in good nutritional condition and did not need nutritional support (scores of 0–3) while over one-third (35.3%) were scored with mild/moderate malnutrition (scores of4–8) and needed to be given nutritional intervention. Nearly half of the patients (45.1%) were in a state of severe malnutrition (scores > 9) and urgently needed nutritional support.

In our research 1867 patients (PG-SGA scores of ≥ 4) required nutritional intervention, but we found only 880 cases (37.9%) that had accepted nutritional support a week before the survey. We found that 1103/1867 (59.1%) of patients needed nutritional intervention but went without nutritional support therapy and 116 well-nourished patients (25.5%) were given the nutritional support treatment (Table 1).

**Table 1:** PG-SGA classification and nutritional therapy situation, *n* = 2322

PG-SGA score	Cases *n* (%)	Nutritional therapy (%)	No nutritional therapy (%)
Not need nutritional support (0 to 3)	455 (19.6)	116 (25.5)	339 (74.5)
Mild/moderate malnutrition(4 to 8)	820 (35.3)	280 (34.1)	540 (65.9)
Severe malnutrition (≥ 9)	1047 (45.1)	484 (46.23)	563 (53.77)

*p* < 0.005

Univariate analysis showed that gender, age, residential area, the proportion of reimbursement, and cultural knowledge were related to the different nutritional groups. Results are summarized in detail in Table 2.

**Table 2:** The influence factors of hospitalized gastric cancer patients’ nutritional status

Variables	The score of PG-SGA			
	0–3	4–8	≥ 9	*p*
Age (years)				
≤ 65	304	481	547	< 0.0001
> 65	151	339	500	
Gender				
Male	334	581	713	0.007
Female	121	239	334	
Residence				
City	258	390	479	
Town	73	152	186	0.004
Village	124	278	382	
Education				
BS or above	32	41	42	
High school	269	471	560	0.002
Primary school or no schooling	154	308	445	
Medical insurance				
Free medical care	230	369	424	
Rural insurance	143	342	461	0.000
Self-paying	82	109	162	

(*p* < 0.05)

We use an ANOVA test to compare NRS2002, BMI, PA, ALB, HB, MAC, TSF, and HGS with the different PG-SGA qualitative evaluations. The differences between nutritional groups were statistically significant *p* < 0.05. As the nutritional status scores became worse, the NRS2002 score increased and the BMI, MAC, TSF, HGS, ALB, and HB scores showed a trend of a gradual decrease, as shown in Table 3.

**Table 3:** Association between the PG-SGA and nutritional parameters

PG-SGA score					
Index	(0–3)	(4–8)	(≥ 9)	F*	*p*
NRS2002 (score)	1.76 ± 1.08	2.59 ± 1.29	3.41 ± 1.26	298.53	< 0.0001
BMI (kg/m^2^)	22.2 ± 3.07	21.4 ± 3.41	20.0 ± 4.54	88.711	< 0.0001
MAC (cm)	25.8 ± 3.25	25.2 ± 3.54	23.9 ± 3.90	50.096	< 0.0001
TSF (mm)	14.91 ± 7.15	14.27 ± 7.80	12.3 ± 6.78	28.056	< 0.0001
HGS (kg)	26.02 ± 13.5	25.4 ± 12.6	21.6 ± 11.2	25.177	< 0.0001
ALB (g/L)	39.73 ± 5.03	37.6 ± 5.14	36.2 ± 12.4	99.745	< 0.0001
Hb (mg/L)	122.6 ± 22.2	117.1 ± 24.8	110.5 ± 31.4	33.265	< 0.0001
KPS (score)	89.9 ± 7.49	85.8 ± 11.75	77.1 ± 16.86	173.245	< 0.0001

*Univariate analysis *p* < 0.05. *BMI*, body mass index; *MAC*, mid-arm diameter; *TSF*, triceps skin-fold; *HGS*, hand-grip strength

Further, using the Spearman rank correlation analysis, we found there was a negative correlation between the PG-SGA quantitative evaluation and BMI, MAC, TSF, HGS, ALB, HB, and KPS. The difference was statistically significant as shown in Table 4.

**Table 4:** Correlation analysis between PG-SGA quantitative evaluation and nutritional parameters, *n* = 2322

	Correlation coefficient^*^	*p*
NRS2002	0.455	< 0.0001
BMI	− 0.267	< 0.0001
MAC	− 0.221	< 0.0001
TSF	− 0.159	< 0.0001
HGS	− 0.165	< 0.0001
ALB	− 0.275	< 0.0001
HB	− 0.207	< 0.0001
KPS	− 0.380	< 0.0001

*Spearman rank correlation coefficient, *p* < 0.05

Considering the relationship between nutritional status and the quality of life, the functional categories and the overall health status score mean were significantly lower while the symptom categories markedly increased in patients with higher PG-SGA scores, *p* < 0.001. As shown in Table 5.

**Table 5:** The correlation of nutritional status and quality of life in patients with gastric cancer

PG-SGA score				
Categories	0–3	4–8	≥ 9	p*
Physical functioning	79.965 ± 23.725	79.933 ± 23.755	79.930 ± 23.753	< 0.0001
Role functioning	74.114 ± 27.465	74.066 ± 27.500	74.060 ± 27.499	< 0.0001
Emotional functioning	84.103 ± 18.554	84.043 ± 18.643	84.036 ± 18.649	< 0.0001
Cognitive functioning	84.889 ± 19.569	84.854 ± 19.643	84.851 ± 19.641	< 0.0001
Social functioning	67.952 ± 26.481	67.919 ± 26.523	67.913 ± 26.526	< 0.0001
Global QOL	57.796 ± 20.417	57.750 ± 20.462	57.736 ± 20.459	< 0.0001
Fatigue	24.206 ± 22.914	24.238 ± 22.960	24.256 ± 22.951	< 0.0001
Nausea/vomiting	10.489 ± 18.824	10.554 ± 18.981	10.557 ± 15.233	< 0.0001
Pain	17.532 ± 22.239	17.583 ± 22.309	17.586 ± 22.307	< 0.0001
Dyspnea	9.373 ± 18.578	9.328 ± 18.615	9.309 ± 18.606	< 0.0001
Insomnia	20.180 ± 25.073	20.182 ± 25.105	20.188 ± 25.101	< 0.0001
Appetite loss	20.296 ± 26.264	20.284 ± 26.293	20.275 ± 26.285	< 0.0001
Constipation	10.116 ± 20.308	10.180 ± 20.282	10.191 ± 20.283	< 0.0001
Diarrhea	5.471 ± 15.100	5.510 ± 15.233	5.536 ± 15.233	< 0.0001
Financial problems	33.973 ± 30.050	34.029 ± 30.077	34.014 ± 30.792	< 0.0001

*Kruskal-Wallis tests, *p* < 0.01

## Discussion

Gastric cancer is one of the most common malignant tumors in China. Surgery and chemoradiotherapy are the main anti-tumor treatments. The presence of the tumor and its treatment might aggravate the patient’s nutritional status. Studies have shown that malnutrition will reduce the quality of life [15] and encourage treatment resistance. It will also increase the risk of infection, the incidence of postoperative complications, and the mortality rate [16]. It is important to identify patients with malnutrition or who are at risk of developing malnutrition in a timely manner and to provide necessary nutritional support. It is beneficial to promote recovery and improve prognosis [17]. The PG-SGA was modified based on subjective global assessment (SGA) by Ottery. It was developed especially as a malignant tumor patients’ nutritional screening tool. The American Dietetic Association recommended it as the nutrition evaluation standard for malignant tumor patients, but it has had few applications in China.

The incidence of malnutrition varies among different kinds of malignant tumors; generally, patients with head and neck cancer or digestive tract malignant tumors are at a higher risk for malnutrition than patients with other types of tumors [18]. According to the results of our study, 80.4% of hospitalized gastric cancer patients were found to have PG-SGA scores of ≥ 4 and 45.1% of patients had severe malnutrition, PG-SGA ≥ 9. This is similar to the findings of Liyan Zhang [19]. In his report, the majority of hospital patients with advanced gastrointestinal cancer were malnourished and nearly half of the patients were severely malnourished and needed nutritional support before anti-tumor treatment. Their results support our claim that malnutrition is very common in gastric cancer patients. Patients with gastric cancer have difficulty eating and digesting. There can be inadequate intake of energy because of pyloric obstruction and tumor-associated factors cause a profound effect on fat metabolism and protein synthesis. In addition, adverse reactions to anticancer treatment, such as nausea, vomiting, fatigue, and pain, can also lead to the deterioration of the patient’s nutritional status. For some postoperative gastric cancer patients, surgical complications or function reconstruction can also lead to malnutrition [20–22]. In addition, social and psychological factors may affect the nutritional status of patients.

According to the survey, nutrition support treatment for gastric cancer patients is not always possible [23, 24]. In our study, 59.1% of malnourished gastric cancer patients (1103/1867) did not receive any treatment and 25.5% of patients (116/455) with good nutrition were given nutritional support. This unreasonable situation is very common in some big hospitals in China [4, 25, 26]. It is urgent to revise, standardize, and popularize practical and feasible guidelines for nutritional support in the whole country.

Studies find that poor nutrition has a negative impact on cancer patients, such as weight loss that can lead to fatigue and the deterioration of anorexia, the patients’ survival rate drops, anti-tumor tolerance is reduced, and complications and side effects increase. Therefore, the medical staff should pay more attention to and educate on the subject of malnutrition in gastric cancer patients. The staff needs to be timely to assess the nutritional status and provide reasonable nutritional intervention/therapy for malnourished patients to improve the patients’ quality of life and clinical outcome.

Univariate analysis showed gastric cancer malnutrition was related to the patients’ gender and age. Females were more likely to present with severe malnutrition, and this is consistent with the results from Yangping [27]. The reason is likely related to the female patients’ psychological factors such as anxiety, depression, fear, eating less, and a worse immune function. Liyan Zhang [19] also confirmed a worse nutritional status in elderly gastric cancer patients. A Korean study [28] suggests more postoperative malnutrition in elderly patients. That is to say that elderly patients have more basic diseases along with worse gastrointestinal consumption and absorption function, and malnutrition would be more likely in these gastric cancer patients. More attention should be paid to these patients. The nutritional state of patients who lived in rural areas had less education and was burdened with more hospitalization expenses which were also worse. So patient nutrition education is necessary, and the government should further improve the serious illness medical insurance policy, improve the reimbursement ratio, and encourage patients to participate in commercial medical insurance in order to improve security.

Currently, NRS2002 and PG-SGA are the most widely used for nutritional risk screening evaluations [29], but they are still not the gold standard for the world. NRS2002 has its shortcomings, such as it is difficult to measure accurate weight when patients cannot get out of bed, or if they have edema or ascites, and its use will be limited. The nutritional assessment tool PG-SGA, with good sensitivity and specificity, is the most ideal and widely used nutritional assessment tool and has good consistency with other tools [30, 31]. It is recommended for a variety of malignant tumors in Europe and the USA, such as digestive tract tumor, head and neck cancer, and gynecologic tumors [32–34].

In comparison with NRS2002, we determined BMI, ALB, Hb, MAC, TSF, and HGS and we found that PG-SGA had good consistency with these nutritional parameters, and among the different PG-SGA scores, the differences were statistically significant. When the nutritional status was worse, the NRS2002 score increased and the results from BMI, PA, ALB, Hb, MAC, TSF, and HGS showed a decreasing trend. The PG-SGA evaluation was in accord with another nutritional assessment and was suitable for patients with malignant tumors, and the assessment is worthy of clinical popularization and application.

The QLQ-C30 was produced by The European Organization for Research and Treatment of Cancer (EORTC) and has been widely adopted in many countries to investigate the quality of life for cancer patients [35, 36]. QLQ-C30 is known to work in China [11, 37]. By the Kruskal-Wallis test, we found that as the PG-SGA score was increasing, values from the functional category and for the overall health status of patients with a lower mean field rank and the symptoms category rank mean increased. It turned out that as the functional abilities and the quality of life become worse, symptoms or problems, such as fatigue, nausea and vomiting, loss of appetite, and insomnia, become worse and add to the poor quality of life. It was also confirmed that the nutritional status was related to the patients’ economic situation.

There are limitations to the research. The malnourished patients were without further nutritional intervention and we are hoping to clarify in future research whether an improvement in the nutritional status in gastric cancer patients will produce a better clinical outcome. In addition, the effect of nutritional status on the final clinical outcome after nutritional therapy was not followed up.

In a word, malnutrition is common in patients with gastric cancer and has a significant impact on the quality of life. We should pay full attention at the time of clinical diagnosis and treatment and screen for the presence of malnourished patients, provide timely and reasonable nutritional intervention to enhance their tolerance of anti-tumor therapy, and improve the patients’ quality of life.

### Source of funding

The National Key Research and Development Program (No.: 2017YFC1309200).

## References

[1] Wan Qing C, Rong Shou Z, Baade PD, Si Wei Z, Hong Mei Z, Bray F (2016). Cancer statistics in China, 2015. CA Cancer J Clin.

[2] Thoresen L, Fieldstad I, Kroqstad K, Kaasa S, Falkmer UG (2002). Nutritional status of patients with advanced cancer: the value of using the subjective global assessment of nutritional status as a screening tool. Palliat Med.

[3] Wie GA, Cho YA, Kim ST, Kim SM, Bae JM, Joung H (2010). Prevalence and risk factors of malnutrition and stage in the National Cancer Center in Korea. Nutrition..

[4] Zhu Ming J, Wei C, Sai Nan ZA (2008). Survey on the prevalence of malnutrition (deficiency), the incidence of nutritional risk and nutritional support in the top three hospitals in the east, middle and west of China. Chinese J of Clin Nutr.

[5] Pressor M, Desne S, Berchery D, Rossignol G, Poiree B, Meslier M (2010). Prevalence, risk factors and clinical implications of malnutrition in French Comprehensive Cancer Centers. Br J Can.

[6] Von Meyenfeldt M (2005). Caner-associated malnutrition: an introduction. Eur J Oncol Nurs.

[7] Tchekmedya SN, Zahyna D, Halpert C, Heber D (1992). Assessment and maintenance of nutrition in older cancer patients. Oncology..

[8] Ottery FD (1994). Rethinking nutritional support of the cancer patient the new field of nutritional oncology. Semin Oncol.

[9] Kondrup J, Allison SP, Elia M, Vellas B, Plauth M (2002). Educational and Clinical Practice Committee, European Society of Parenteral and Enteral Nutrition (ESPEN). ESPEN guidelines for nutrition screening. ClinNutr.

[10] Kondrup J (2003). Nutritional risk screening (NRS 2002): a new method based on an analysis of controlled clinical trials. Clin Nutr Jun.

[11] Wan C, Chen M, Zhang C (2005). Comment to the Chinese version of the quality of life instrument EORTC QLQ-30. J Pract Oncol.

[12] Jelliffe DB (1996) The assessment of nutrition status of the community. World Health Organization Monograph Series No. 53, Geneva, 50–84

[13] Frisancho AR (2008). Anthropometric standards. An interactive nutritional reference of body size and body composition for children and adults.

[14] Schlüssel MM, dos Anjos LA, de Vasconcellos MT, Kac G (2008). Reference values of handgrip dynamometry of healthy adults: a population-based study. Clin Nutr.

[15] Gupta D, Lis CG, Granick J, Grutsch JF, Vashi PG, Lammersfeld CA (2006). Malnutrition was associated with poor quality of life in colorectal cancer: a retrospective analysis. J Clin Epidem.

[16] Correia MI, Campos ACELAN (2003). Cooperative study prevalence of hospital malnutrition in Latin America: the multicenter ELAN study. Nutrition..

[17] Guo hao W (2002). The causes and harms of malnutrition in cancer patients. China J Practical Surg.

[18] Hong Ming P, San Jun C, Jia Fu J, Zhi Wei J, Hou Jie L, Feng L (2013). The impact of nutritional status, nutritional risk, and nutritional treatment on clinical outcome of 2248 hospitalized cancer patients: a multi-center, prospective cohort study in Chinese teaching hospitals. Nutr Cancer.

[19] Liyan Z, Yuhan L, Yu F (2014). Nutritional status and related factors of patients with advanced gastrointestinal cancer. Br J of Nutr.

[20] Yiqiong Y (2011). Investigation and analysis of nutritional risk screening in patients with gastric cancer. West China Med J.

[21] Van Cutsem E, Arends J (2005). The causes and consequences of cancer-associated malnutrition. Eur J Oncol Nurs.

[22] Hébuterne X, Lemarié E, Michallet M, de Montreuil CB, Schneider SM, Goldwasser F (2014). Prevalence of malnutrition and current use of nutrition support in patients with cancer. J Parenter Enter Nutr.

[23] Lidia S, Franco C, Fabrizio P (2011). Nutritional screening and early treatment of malnutrition in cancer patients. J Cachexia Sarcopenia Muscle.

[24] Agostino P, Malattie UO (2011). Metabolic nutrition intervention for improving treatment tolerance in cancer patient. Curr Opin Oncol.

[25] Jiang ZM, Chen W, Zhu SN (2007). Parenteral and enteral nutrition application in west, middle and east China: a multi-center investigation for 15,098 patients in 13 metropolitans using Nutritional Risk Screening 2002 tool. Clin Nutr.

[26] Zi Jian L, Yu Xing Y, Hai Long L, Qin Bing Y, Wei C (2016). Analysis of tumor associated malnutrition in hospitalized patients: a cross-sectional study. Chinese J Clinicians.

[27] Ping Y, Bo C, Xiao TW (2011). Nutritional risk and clinical nutritional support therapy of hospitalized gastric cancer. Surv Chin Surg Found Clin J.

[28] Shim H, Cheong JH, Lee KY, Lee H, Lee JG, Noh SH (2013). Perioperative nutritional status changes in gastrointestinal cancer patients. Yonsei Med.

[29] Bauer J, Capra S, Ferguson M (2002). Use of the scored patient-generated subjective global assessment (PG-SGA) as a nutrition assessment tool in patients with cancer. Eur J of Clin Nutr.

[30] Luo Q, Cao WX (2010). Evaluation of nutritional status of patients with digestive system malignant tumor by different nutritional evaluation methods. J Surg Theory Pract.

[31] Carolina A, Rosa COB, Ribeiro AQ, Ribeiro CAR (2015). Patient-generated subjective global assessment and classic anthropometry: comparison between the methods in detection of malnutrition among elderly with cancer. Nutr Hosp.

[32] Phippen NT, Lowery WJ, Barnett JC, Hall LA, Landt C, Leath CA (2011). Evaluation of the patient generated subjective global assessment (PG-SGA) as a predictor of febrile neutropenia in gynecologic cancer patients receiving combination chemotherapy a pilot study. Gynecol Oncol.

[33] Correira Pereira MA, Santos CA, Almeida Brito J, Fonseca J (2014). Scored patient-generated subjective global assessment, albumin and transferrin for nutritional assessment of gastrostomy fed head or neck cancer patients. Nutr Hosp.

[34] Hill A, Kiss N, Hodgson B, Crowe TC, Walsh AD (2011). Associations between nutritional status weight loss radiotherapy treatment toxicity and treatment outcomes in gastrointestinal cancer patients. Clin Nutr.

[35] Schwarz R, Hinz A (2001). Reference data for the quality of life questionnaire EORTC QLQ-C30 in the general German population. Eur J Cancer.

[36] Derogar M, Van SM, Lagergren P (2012). Reference values for the EORTC QLQ-C30 quality of life questionnaire in a random sample of the Swedish population. Acta Oncol.

[37] Jun Mei S, Xiao Xiang W, Ying L, Chun Yi L, Dan Dan C (2015). Reliability and validity of EORTC QLQ-C30 in postoperative chemotherapy for gastric carcinoma patients. J Hebei Med Univ.

